# Supplementary material for: Fatty Acid Composition of Cosmetic Argan Oil: Provenience and Authenticity Criteria
Source: Molecules. 2020 Sep 7;25(18):4080. doi: 10.3390/molecules25184080 (PMC7570657; doi:10.3390/molecules25184080)
Supplement: Supplementary file 1 [file molecules-25-04080-s001.pdf]

Table S1. Time of processing, geoenvironmental and climatic information of the sampling locations together with the isotopic composition of hydrogen and oxygen in precipitation.

Province of the sample collection	Time of proces.	Lat	Log	Altitude	Dist. from the coast	rain $\delta^{18}O$	rain $\delta^{2}H$	T	rain	Rel. hum.	Aridity Index
				m	km	‰	‰	°C	mm	°F	
Chtouka ait baha	Apr-2017	30.075	−9.046	542	59	−3.7	−15	21.4	235	47.2	7.50
Chtouka ait baha	Nov-2018	30.011	−9.412	542	28	−4.8	−23	21.4	235	47.2	7.50
Chtouka ait baha	May-2018	30.011	−9.412	542	29	−3.9	−17	21.4	235	47.2	7.50
Chtouka ait baha	Nov-2018	30.528	−9.333	684	31	−4.1	−18	20.6	277	46.2	9.06
Chtouka ait baha	Apr-2018	30.528	−9.333	684	31	−4.1	−18	20.6	277	46.2	9.06
Chtouka ait baha	Nov-2018	30.027	−9.235	542	42	−4.7	−22	21.4	235	47.2	7.50
Chtouka ait baha	May-2018	30.027	−9.235	542	42	−4.7	−22	21.4	235	47.2	7.50
Chtouka ait baha	May-2018	30.027	−9.235	542	43	−4.7	−22	21.4	235	47.2	7.50
Chtouka ait baha	Dec-2018	30.093	−9.157	542	49	−4.8	−23	21.4	235	47.2	7.50
Chtouka ait baha	May-2018	30.093	−9.157	542	49	−4.8	−23	21.4	235	47.2	7.50
Essaouira	Nov-2018	31.519	−9.450	317	26	−4.4	−21	19.9	297	98.1	9.92
Essaouira	Dec-2018	31.539	−9.546	139	16	−4.0	−18	19.3	271	100	9.23
Essaouira	Nov-2018	31.555	−9.608	139	11	−4.0	−18	19.3	271	100	9.23
Essaouira	Jan-2019	31.536	−9.457	317	24	−4.5	−21	19.9	297	98.1	9.92
Essaouira	Dec-2018	31.551	−9.475	317	24	−4.4	−21	19.9	297	98.1	9.92
Essaouira	Dec-2018	31.282	−9.719	310	9	−4.4	−21	19.6	264	63.3	8.93
Essaouira	Dec-2018	31.409	−9.673	310	15	−4.4	−21	19.6	264	63.3	8.93
Essaouira	Jan-2019	31.282	−9.719	310	9	−4.4	−21	19.6	264	63.3	8.93
Essaouira	Nov-2018	31.282	−9.719	310	9	−4.2	−18	19.6	264	63.3	8.93
Essaouira	Dec-2018	31.282	−9.719	310	9	−4.2	−18	19.6	264	63.3	8.93
Essaouira	Jan-2019	31.282	−9.719	310	9	−4.2	−18	19.6	264	63.3	8.93
Essaouira	Oct-2018	31.282	−9.719	310	9	−4.2	−18	19.6	264	63.3	8.93
Essaouira	Dec-2018	30.633	−9.884	299	2	−4.2	−18	19.6	264	63.3	8.93
Essaouira	May-2018	31.002	−9.683	310	13	−4.4	−20	19.6	264	63.3	8.93
Essaouira	Jan-2019	31.225	−9.653	310	17	−5.2	−26	19.6	264	63.3	8.93
Essaouira	Mar-2018	31.225	−9.653	310	17	−5.2	−26	19.6	264	63.3	8.93
Essaouira	Jan-2019	31.551	−9.475	317	23	−4.4	−21	19.9	297	60.3	9.92
Essaouira	Dec-2018	31.568	−9.473	317	22	−4.4	−21	19.9	297	60.3	9.92

Essaouira	May-2018	31.554	−9.302	317	38	−4.8	−23	19.9	297	60.3	9.92
Essaouira	Mar-2018	31.082	−9.749	310	8	−4.4	−22	19.6	264	63.3	8.93
Essaouira	Nov-2018	31.277	−8.894	240	35	−4.4	−21	20.8	314	58.9	10.19
Essaouira	Jan-2019	31.536	−9.457	317	24	−4.5	−21	19.9	297	60.3	9.92
Essaouira	Dec-2018	31.554	−9.302	319	24	−4.5	−21	19.9	297	60.3	9.92
Sidi Ifni	Jan-2019	29.195	−10.13	251	17	−3.8	−16	20.9	156	63.5	5.05
Sidi Ifni	Jan-2019	29.336	−9.642	536	47	−6.0	−31	21.5	163	49.7	5.15
Sidi Ifni	Apr-2018	29.336	−9.642	537	47	−6.0	−31	21.5	163	49.7	5.15
Sidi Ifni	Dec-2018	29.195	−10.13	251	17	−3.8	−16	20.9	156	63.5	5.05
Sidi Ifni	Jan-2019	29.361	−9.732	536	38	−5.9	−30	21.5	163	49.7	5.15
Sidi Ifni	Apr-2018	29.361	−9.732	537	38	−5.8	−29	21.5	163	49.7	5.15
Sidi Ifnl	Dec-2018	29.195	−10.13	251	20	−3.8	−16	20.9	156	63.5	5.05
Taroudante	Jan-2019	30.754	−8.515	1114	124	−5.8	−30	19.5	248	39.8	8.42
Taroudante	Mar-2018	30.754	−8.515	1114	124	−5.8	−30	19.5	248	39.8	8.42
Taroudante	Dec-2018	30.379	−8.694	1035	87	−4.7	−22	20.1	201	39.1	6.68
Taroudante	Apr-2018	30.379	−8.694	1035	87	−4.7	−22	20.1	201	39.1	6.68
Taroudante	Jan-2019	30.705	−8.886	1035	80	−5.9	−31	19.6	248	39.8	8.40
Taroudante	Apr-2018	30.705	−8.886	1114	85	−6.9	−38	19.6	248	39.8	8.40
Taroudante	Jan-2019	30.304	−8.487	1270	109	−6.3	−33	19.3	182	36.0	6.22
Taroudante	Nov-2018	30.304	−8.487	1270	109	−6.3	−33	19.3	182	36.0	6.22
Taroudante	Apr-2018	30.304	−8.487	1270	109	−6.3	−33	19.3	182	36.0	6.22
Taroudante	Sep-2018	30.304	−8.487	1270	109	−6.3	−33	19.3	182	36.0	6.22
Taroudante	Jan-2019	30.379	−8.694	1035	87	−4.7	−22	20.1	201	39.1	6.68
Taroudante	Feb-2018	30.379	−8.694	1035	87	−4.7	−22	20.1	201	39.1	6.68
Taroudante	Jan-2019	30.506	−8.609	1114	100	−4.4	−20	19.6	248	39.8	8.40
Taroudante	May-2018	30.506	−8.609	1114	100	−4.4	−20	19.6	248	39.8	8.40
Taroudante	Dec-2018	30.609	−9.077	648	59	−4.3	−20	20.6	277	46.2	9.06
Taroudante	Apr-2018	30.609	−9.077	648	59	−4.3	−20	20.6	277	46.2	9.06
Tiznite	Jan-2019	30.616	−9.349	309	8	−4.1	−18	19.6	264	63.3	8.93
Tiznite	Apr-2018	31.282	−9.719	309	8	−4.1	−18	19.6	264	63.3	8.93
Tiznite	Dec-2018	29.631	−9.389	784	50	−4.8	−22	20.8	207	45.5	6.71
Tiznite	Feb-2018	29.631	−9.389	784	50	−4.8	−22	20.8	207	45.5	6.71
Tiznite	Mar-2018	29.631	−9.389	784	49	−4.8	−22	20.8	207	45.5	6.71
Tiznite	Nov-2018	29.631	−9.389	542	59	−3.7	−15	21.4	235	47.2	7.50
Tiznite	Dec-2018	29.463	−9.664	536	42	−5.3	−26	21.5	163	49.7	5.15
Tiznite	May-2018	29.463	−9.664	536	42	−5.3	−26	21.5	163	49.7	5.15

Tiznite	Jan-2019	29.601	−9.906	393	11	−4.2	−18	20.6	205	58.4	6.71
Tiznite	Apr-2018	29.601	−9.906	393	11	−4.2	−18	20.6	205	58.4	6.71

Table S2. Acidity and fatty acid composition (in wt. % of total FA) of argan oils from different sampling locations.

Reagion of the sample	Acidity	C14:0	C16:0	C16:1	C18:0	C18:1	C18:2	C18:3	C20:0	C20:1	C22:0	C22:1	C24:0	C18:1 T	C18:2 CT	C18:3 CTC	C18:2 CT + C18:3 CTC
	wt %									wt. % of total FA							
Agadir Idaw Tanane	0.53	0.13	12.76	0.13	5.74	48.82	31.25	0.17	0.35	0.38	0.10	0.02	0.05	0.02	0.02	0.07	0.09
Chtouka ait baha	0.43	0.15	14.07	0.13	6.54	47.17	30.74	0.15	0.39	0.35	0.12	0.01	0.07	0.02	0.02	0.06	0.08
Chtouka ait baha	0.44	0.13	13.68	0.13	7.02	43.79	34.03	0.17	0.43	0.33	0.11	0.02	0.06	0.02	0.02	0.06	0.08
Chtouka ait baha	0.49	0.13	13.16	0.12	6.81	43.20	35.32	0.17	0.43	0.34	0.14	0.01	0.07	0.02	0.03	0.06	0.09
Chtouka ait baha	1.41	0.16	13.38	0.11	6.48	47.06	31.58	0.17	0.40	0.35	0.12	0.02	0.06	0.02	0.02	0.06	0.09
Chtouka ait baha	0.68	0.15	12.99	0.12	6.42	46.53	32.58	0.17	0.40	0.35	0.12	0.02	0.06	0.02	0.02	0.06	0.08
Chtouka ait baha	0.71	0.14	12.74	0.12	6.13	45.88	33.83	0.17	0.37	0.35	0.11	0.02	0.05	0.02	0.02	0.06	0.08
Chtouka ait baha	0.44	0.15	13.25	0.13	6.46	46.07	32.74	0.16	0.41	0.34	0.12	0.01	0.06	0.02	0.02	0.06	0.08
Chtouka ait baha	0.48	0.14	12.74	0.11	6.43	44.07	35.26	0.17	0.40	0.34	0.13	0.02	0.06	0.02	0.03	0.06	0.09
Chtouka ait baha	0.32	0.14	13.17	0.12	6.61	45.43	33.35	0.17	0.40	0.33	0.11	0.01	0.06	0.02	0.02	0.06	0.08
Chtouka ait baha	0.73	0.14	12.86	0.11	6.73	43.52	35.34	0.18	0.43	0.34	0.15	0.01	0.07	0.03	0.03	0.06	0.09
Essaouira	0.35	0.14	13.62	0.15	5.84	50.41	28.65	0.15	0.37	0.38	0.11	0.01	0.06	0.03	0.02	0.06	0.08
Essaouira	5.45	0.13	11.98	0.11	5.74	45.44	35.37	0.17	0.38	0.37	0.13	0.02	0.06	0.02	0.02	0.06	0.09
Essaouira	1.15	0.12	11.50	0.12	5.80	43.40	37.92	0.18	0.34	0.34	0.10	0.01	0.04	0.02	0.02	0.06	0.08
Essaouira	1.46	0.15	13.27	0.08	6.51	39.46	39.32	0.20	0.38	0.36	0.09	0.01	0.04	0.02	0.02	0.06	0.08
Essaouira	0.49	0.16	13.80	0.15	5.45	48.11	31.21	0.14	0.34	0.37	0.10	0.02	0.05	0.03	0.02	0.06	0.08
Essaouira	0.79	0.11	12.21	0.11	5.98	46.18	34.23	0.18	0.38	0.34	0.10	0.02	0.05	0.02	0.02	0.06	0.09
Essaouira	3.04	0.14	12.65	0.11	5.55	48.34	32.03	0.17	0.36	0.37	0.11	0.02	0.05	0.02	0.02	0.06	0.08
Essaouira	0.88	0.14	12.45	0.11	5.62	48.85	31.66	0.16	0.35	0.39	0.11	0.02	0.05	0.03	0.02	0.06	0.08
Essaouira	0.54	0.14	12.88	0.12	5.66	46.97	33.07	0.16	0.35	0.36	0.11	0.02	0.05	0.02	0.02	0.06	0.08
Essaouira	1.10	0.14	12.76	0.13	5.68	47.60	32.54	0.16	0.36	0.37	0.10	0.02	0.05	0.02	0.02	0.06	0.08
Essaouira	0.58	0.14	12.77	0.12	6.66	44.44	34.65	0.17	0.42	0.32	0.12	0.01	0.06	0.02	0.02	0.06	0.08
Essaouira	0.60	0.14	12.95	0.13	6.12	46.33	33.08	0.17	0.40	0.37	0.13	0.02	0.07	0.02	0.02	0.06	0.08
Essaouira	0.86	0.13	12.67	0.13	5.62	48.12	32.14	0.16	0.36	0.38	0.11	0.02	0.05	0.02	0.02	0.06	0.09
Essaouira	0.49	0.14	12.73	0.12	5.92	46.61	33.28	0.17	0.38	0.37	0.12	0.02	0.06	0.02	0.02	0.06	0.08
Essaouira	0.38	0.13	12.31	0.12	5.56	46.50	34.21	0.17	0.35	0.37	0.11	0.02	0.05	0.02	0.02	0.06	0.08
Essaouira	0.38	0.13	12.47	0.12	5.79	46.29	34.04	0.17	0.36	0.36	0.11	0.02	0.05	0.02	0.02	0.06	0.09
Essaouira	0.75	0.15	12.87	0.13	5.81	48.42	31.37	0.16	0.38	0.41	0.13	0.02	0.06	0.02	0.02	0.07	0.09

Essaouira	0.42	0.10	13.47	0.14	5.84	46.93	32.33	0.16	0.36	0.39	0.11	0.01	0.05	0.02	0.02	0.07	0.09
Essaouira	0.38	0.15	13.20	0.13	5.68	47.44	32.23	0.16	0.35	0.38	0.11	0.02	0.06	0.02	0.02	0.06	0.08
Essaouira	0.33	0.13	12.86	0.13	5.27	48.77	31.68	0.15	0.33	0.39	0.12	0.01	0.06	0.02	0.02	0.06	0.09
Essaouira	0.36	0.16	13.08	0.12	5.82	47.13	32.53	0.16	0.36	0.38	0.10	0.02	0.05	0.02	0.02	0.06	0.08
Essaouira	0.26	0.12	12.57	0.12	5.81	44.96	35.25	0.18	0.37	0.33	0.11	0.02	0.06	0.02	0.02	0.06	0.08
Essaouira	0.39	0.14	12.71	0.12	5.65	46.92	33.26	0.16	0.35	0.38	0.12	0.01	0.06	0.02	0.02	0.06	0.08
Sidi Ifni	1.07	0.14	13.82	0.14	6.82	50.78	27.07	0.14	0.43	0.36	0.12	0.01	0.07	0.02	0.02	0.07	0.09
Sidi Ifni	0.38	0.15	14.76	0.16	7.23	47.81	28.63	0.14	0.48	0.32	0.13	0.01	0.08	0.02	0.02	0.06	0.09
Sidi Ifni	0.55	0.14	13.64	0.16	6.45	48.74	29.64	0.15	0.43	0.34	0.14	0.01	0.07	0.03	0.02	0.06	0.08
Sidi Ifni	0.54	0.14	13.83	0.16	6.62	47.57	30.48	0.15	0.43	0.32	0.12	0.01	0.07	0.02	0.02	0.06	0.08
Sidi Ifni	0.49	0.15	12.54	0.12	6.83	44.49	34.62	0.17	0.43	0.32	0.14	0.01	0.06	0.02	0.02	0.06	0.08
Sidi Ifni	0.22	0.14	13.55	0.16	6.53	46.50	31.88	0.15	0.45	0.32	0.14	0.01	0.07	0.03	0.02	0.06	0.08
Sidi Ifnl	0.42	0.14	14.21	0.14	6.90	47.74	29.52	0.15	0.49	0.36	0.16	0.01	0.09	0.03	0.02	0.06	0.09
Taroudante	0.32	0.14	13.36	0.13	5.36	50.72	29.20	0.15	0.31	0.37	0.09	0.02	0.05	0.03	0.02	0.06	0.08
Taroudante	0.47	0.14	12.61	0.12	5.74	46.07	34.13	0.17	0.36	0.38	0.12	0.02	0.06	0.03	0.02	0.06	0.09
Taroudante	0.94	0.15	13.51	0.14	6.65	46.38	31.95	0.15	0.43	0.31	0.13	0.01	0.07	0.03	0.02	0.06	0.08
Taroudante	0.87	0.14	12.67	0.12	6.88	44.31	34.65	0.17	0.43	0.32	0.13	0.01	0.06	0.03	0.02	0.06	0.08
Taroudante	0.48	0.14	13.14	0.12	6.33	46.60	32.45	0.16	0.39	0.37	0.11	0.02	0.06	0.03	0.02	0.07	0.09
Taroudante	0.72	0.15	12.48	0.10	6.29	44.22	35.46	0.17	0.40	0.38	0.15	0.02	0.07	0.03	0.03	0.06	0.09
Taroudante	0.50	0.15	12.96	0.11	6.21	46.53	32.82	0.16	0.39	0.38	0.12	0.02	0.06	0.03	0.02	0.06	0.09
Taroudante	0.37	0.14	13.06	0.12	6.17	45.78	33.51	0.16	0.39	0.36	0.12	0.02	0.06	0.03	0.02	0.06	0.09
Taroudante	0.32	0.13	13.15	0.12	6.37	46.47	32.55	0.15	0.41	0.35	0.12	0.02	0.06	0.03	0.02	0.06	0.09
Taroudante	0.32	0.15	14.16	0.14	5.90	46.33	32.09	0.16	0.39	0.36	0.13	0.01	0.07	0.02	0.02	0.06	0.08
Taroudante	0.73	0.15	13.71	0.15	6.52	48.22	30.05	0.14	0.42	0.33	0.12	0.01	0.07	0.02	0.02	0.06	0.08
Taroudante	0.60	0.15	13.41	0.15	6.39	47.39	31.33	0.15	0.41	0.33	0.12	0.01	0.06	0.02	0.02	0.06	0.08
Taroudante	0.66	0.14	13.45	0.13	5.60	48.86	30.69	0.15	0.34	0.36	0.10	0.02	0.05	0.02	0.02	0.06	0.08
Taroudante	0.93	0.13	12.76	0.12	5.91	45.85	33.92	0.21	0.40	0.37	0.14	0.02	0.07	0.02	0.02	0.06	0.08
Taroudante	0.71	0.13	12.40	0.13	6.08	45.02	35.08	0.16	0.38	0.34	0.12	0.02	0.06	0.02	0.02	0.06	0.08
Taroudante	6.54	0.13	12.20	0.13	6.11	44.68	35.52	0.17	0.41	0.34	0.14	0.02	0.06	0.02	0.02	0.06	0.08
Tiznite	0.43	0.13	12.63	0.11	5.59	48.09	32.21	0.18	0.39	0.39	0.12	0.02	0.06	0.02	0.02	0.06	0.08
Tiznite	0.38	0.14	12.99	0.13	6.27	46.18	33.05	0.16	0.41	0.36	0.13	0.02	0.07	0.02	0.02	0.06	0.08
Tiznite	0.77	0.13	13.36	0.14	6.82	45.90	32.46	0.15	0.43	0.32	0.12	0.01	0.07	0.02	0.02	0.06	0.08
Tiznite	0.55	0.14	13.57	0.14	6.59	46.31	31.99	0.15	0.44	0.34	0.14	0.01	0.07	0.02	0.02	0.06	0.08
Tiznite	1.52	0.13	13.57	0.13	6.28	47.44	31.23	0.14	0.40	0.35	0.12	0.01	0.07	0.02	0.02	0.06	0.09
Tiznite	0.48	0.14	15.20	0.17	6.50	47.95	28.82	0.14	0.43	0.33	0.12	0.01	0.08	0.02	0.02	0.06	0.08
Tiznite	0.61	0.16	14.03	0.15	6.43	47.67	30.28	0.14	0.45	0.36	0.14	0.01	0.08	0.02	0.02	0.06	0.08

Tiznite	0.38	0.14	13.22	0.14	6.68	45.89	32.70	0.16	0.43	0.32	0.13	0.01	0.07	0.02	0.02	0.06	0.08
Tiznite	0.49	0.17	14.87	0.17	6.27	48.20	29.14	0.15	0.43	0.31	0.12	0.01	0.07	0.02	0.02	0.06	0.08
Tiznite	0.22	0.14	12.61	0.12	5.49	46.97	33.50	0.16	0.35	0.38	0.11	0.02	0.05	0.02	0.02	0.06	0.08
